# Adhesive Cementation of CAD/CAM Silica-based Ceramics: Effect of Adhesive Type and Long-term Aging on the Bond Strength to Composite Cement

**DOI:** 10.3290/j.jad.b5341383

**Published:** 2024-05-21

**Authors:** Nathalia Ramos da Silva, Evelynn Crhistyann Medeiros Duarte, Dayanne Monielle Duarte Moura, Nathália de Carvalho Ramos, Karina Barbosa Souza, Fábio Roberto Dametto, Mutlu Özcan, Marco Antonio Bottino, Rodrigo Othávio de Assunção e Souza

**Affiliations:** a Dentist, Federal Institute University of Rio Grande do Norte (IFRN), João Câmara Campus, João Câmara/RN, Brazil. Idea, performed the experiments in partial fulfillment of requirements for a degree, wrote the manuscript; consulted on and performed statistical evaluation; contributed substantially to discussion.; b Dentist at Dental Reference Center Dr. Morton Mariz, Ribeira, Natal/RN, Brazil. Wrote the manuscript; consulted on and performed statistical evaluation.; c Professor, State University of Rio Grande do Norte (UERN), Department of Dentistry, Centro, Caicó/RN, Brazil. Idea, hypothesis, experimental design, proofread the manuscript, consulted on and performed statistical evaluation.; d Professor, São Francisco University, Department of Dentistry, Jardim Sao Jose, Bragança Paulista/SP, Brazil. Proofread the manuscript, performed a certain test.; e MSc Student at Department of Dental Materials and Prosthodontics, Institute of Science and Technology, Univ Estadual Paulista (UNESP), Sao Jose dos Campos/SP, 12245-000, Brazil. Proofread the manuscript, performed a certain test.; f Adjunct Professor, Federal University of Rio Grande do Norte (UFRN), Department of Dentistry, Division of Endodontics, Lagoa Nova, Natal/RN, Brazil. Idea, proofread the manuscript.; g Professor, Center for Dental and Oral Medicine, Division of Dental Biomaterials Unit, Clinic for Restorative Dentistry, University of Zürich, Zürich, Switzerland. Idea, proofread the manuscript.; h Titular Professor, Department of Dental Materials and Prosthodontics, Institute of Science and Technology, Univ Estadual Paulista (UNESP), Sao Jose dos Campos/SP, Brazil. Idea, proofread the manuscript.; i Adjunct Professor, Federal University of Rio Grande do Norte (UFRN), Department of Dentistry, Division of Prosthodontics, Lagoa Nova, Natal/RN, Brazil. Idea, hypothesis, proofread the manuscript.

**Keywords:** adhesive, ceramics, dentin-bonding agents, dental materials, surface treatment

## Abstract

**Purpose:**

To investigate the effect of adhesive type and long-term aging on the shear bond strength (SBS) between silica-based ceramics and composite cement (CC).

**Materials and Methods:**

Lithium-silicate (LS), feldspathic (FD) and polymer-infiltrated ceramic (PIC) blocks were sectioned (10 x 12 x 2 mm) and divided into 24 groups considering the factors: “ceramics” (LS, FD, and PIC), “adhesive” (Ctrl: without adhesive; 2SC: 2-step conventional; 3SC: 3-step conventional; 1SU: 1-step universal), and “aging” (non-aged or aged [A]). After the surface treatments, CC cylinders (n = 15, Ø = 2 mm; height = 2 mm) were made and half of the samples were subjected to thermocycling (10,000) and stored in water at 37°C for 18 months. The samples were submitted to SBS testing (100 kgf, 1 mm/min) and failure analysis. Extra samples were prepared for microscopic analysis of the adhesive interface. SBS (MPa) data was analyzed by 3-way ANOVA and Tukey’s test (5%). Weibull analysis was performed on the SBS data.

**Results:**

All factors and interactions were significant for SBS (p<0.05). Before aging, there was no significant difference between the tested groups and the respective control groups. After aging, the LS_1SU (22.18 ± 7.74) and LS_2SC (17.32 ± 5.86) groups exhibited significantly lower SBS than did the LS_Ctrl (30.30 ± 6.11). Only the LS_1SU group showed a significant decrease in SBS after aging vs without aging. The LS_1SU (12.20) group showed the highest Weibull modulus, which was significantly higher than LS_2SC_A (2.82) and LS_1SU_A (3.15) groups.

**Conclusion:**

No type of adhesive applied after silane benefitted the long-term adhesion of silica-based ceramics to CC in comparison to the groups without adhesive.

The evolution of ceramic materials, adhesive cementation, and CAD/CAM (computer-aided design/computer-aided manufacturing) technology has promoted advancements in the field of oral rehabilitation. Several types of silica-based ceramics for CAD/CAM with different microstructures and compositions are commercially available, such as feldspathic ceramics, polymer-infiltrated ceramics, and lithium-silicate with the addition of zirconia; the latter has been more recently introduced on the market.^9,25^ Polymer-infiltrated ceramic is a hybrid ceramic with a dual structure comprised of a sintered ceramic network (75 vol%) infiltrated with a polymeric network (25 vol%).^7^ On the other hand, a zirconia-reinforced glass-ceramic has been developed from lithium-silicate ceramic, and has been reported to combine excellent optical and mechanical properties.^32^

The clinical longevity of ceramic restorations is directly related to the adhesion between ceramic, cement, and dental substrate.^9^ This adhesion is even more relevant in cases where dental preparation may not promote high mechanical retention, such as in dental preparations for laminate veneers, onlay, and overlay. Several studies have reported a satisfactory survival rate for feldspathic ceramic restorations (91% for 499 laminate veneers in a 20-year follow-up, with 17 failures),^12^ polymer-infiltrated ceramics (96.4% for 103 partial restorations in a 3-year follow-up, with 3 failures),^26^ and lithium-silicate (91% for 54 partial crowns in a 5-year follow-up, with 5 failures in the group with 0.5-0.74 mm material thickness).^22^ However, despite satisfactory clinical performance, failures such as debonding and fractures have been reported.^12,22,26^

To reduce the occurrence of these failures, clinical and laboratory studies have investigated different surface treatment protocols for the adhesive cementation of silica-based ceramics. The surface treatment indicated for silica-based ceramics is etching with hydrofluoric acid followed by silane.^1,2,24^ Some manufacturers also indicate applying an adhesive layer after ceramic etching and silanization. However, the positive effect of this adhesive layer has been questioned, considering that a simplified protocol with reduced steps can facilitate the clinical routine and reduce treatment costs.^1,6,18-20,31^

In addition, there are several adhesives with different chemical compositions and clinical indications which can make it difficult for professionals to choose an adhesive. Adhesives can be classified into conventional etch-and-rinse (two or three steps), self-etching (one or two steps), or universal (both adhesive strategies can be applied).^27^ Furthermore, some manufacturers have introduced silane and functional monomers, such as 10-MDP, into the composition of universal adhesives, intending to combine the function of ceramic primer and adhesive in one product.^5^

Previous studies that investigated the effect of adhesive applications on the bond strength between ceramic and composite cement presented divergent findings.^5,17,19,20,31^ Some authors reported that applying an adhesive on etched and silanized glass-ceramics did not improve the resin bond strength,^19,20,31^ indicating that this step may be unnecessary. However, other studies showed higher bond strength and stability when an adhesive layer was applied,^5,17^ which may be beneficial for the longevity of ceramic restorations.

Additionally, the different physicochemical characteristics of these adhesives can impact the bond strength and stability of the adhesion between the silica-based ceramic and the composite cement.^6,29^ Few studies have evaluated the effect of the adhesive type on the bond strength. Some authors reported that a hydrophobic adhesive applied on the ceramic surface promoted more stable adhesion to composite cement than did a hydrophilic adhesive.^4^ Vanderlei et al^29^ reported that a high-pH adhesive (pH = 5.6 ± 0.5) produced greater bond strength than did a low-pH adhesive (pH = 1.7 ± 0.2). On the other hand, Garboza et al^8^ and Romanini-Junior et al^23^ reported that the adhesive type did not affect the bond strength between ceramics and composite cement.

Therefore, considering the scarcity of evidence and lack of consensus on the influence of adhesive type on the long-term bond strength of ceramics to composite cement, the objective of this study was to investigate the effect of different types of adhesives, aging, and bond strength of silica-based ceramics for CAD/CAM to composite cement. The tested hypotheses were: a) adhesive type does not affect the bond strength between the tested ceramics and the composite cement; b) the ceramic type does not influence the bond strength; c) aging does not decrease bond strength, regardless of the adhesive or ceramic type.

## Materials and Methods

Details on the materials used in this study are shown in Table 1.

**Table 1:** Material, trademark, manufacture, batch number (nº), and chemical composition of the materials used

Material	Trademark	Manufacture/batch no.	Chemical composition
Polymer-infiltrated ceramic	Vita Enamic	VITA Zahnfabrik; Bad Säckingen, Germany 41101	SiO_2_, Al_2_O_3_, Na_2_O, K_2_O, B_2_O_3_, CaO, TiO_2_
Feldspathic ceramic	VITA Mark II	VITA Zahnfabrik 16940	SiO_2_, Al_2_O_3_, Na_2_O, K_2_O, CaO, TiO_2_
Lithium-silicate ceramic	Celtra Duo	Dentsply Sirona; Konstanz, Germany 18018772	SiO_2_, P_2_O_5_, Al_2_O_3_, Li_2_O, K_2_O, ZrO, CeO_2_, Na_2_O, Tb_4_O_7_, V_2_O_5_, Pr_6_O_11_, Cr, Cu, Fe, Mg, Mn, Si, Zn, Ti, Zr, Al
Hydrofluoric acid	Condac Porcelana 5%	FGM Produtos Odontológicos; Joinville, SC, Brazil 100222	Hydrofluoric acid 5%, water, thickener, surfactant and colouring
Silane	RelyX Ceramic Primer	3M Oral Care; St Paul, MN, USA N878550	Ethyl alcohol, water, methacryloxypropyltrimethoxysilane*
2-step conventional adhesive	Adper Single Bond 2	3M Oral Care 1916200361	Ethyl alcohol, bisphenol A diglycidyl ether dimethacrylate (bis-GMA), silane treated silica, 2-hydroxyethyl methacrylate (HEMA), copolymer of acrylic and itaconic acids, glycerol 1,3 dimethacrylate, diurethane dimethacrylate (UDMA), water, diphenyliodonium hexafluorophosphate*
3-step conventional adhesive	Scotchbond Multi-Purpose	3M Oral Care 76440NE	Bisphenol A diglycidyl ether dimethacrylate (bis-GMA), 2-hydroxyethyl methacrylate (HEMA), triphenylantimony*
1-step-universal adhesive	Single Bond Universal	3M Oral Care 2104300675	2-hydroxyethyl methacrylate, bisphenol a diglycidyl ether dimethacrylate (bis-GMA), 2-propenoic acid, 2-methyl-, reaction products with 1,10-decanediol and phosphorus oxide (P_2_O_5_), ethanol, water, silane-treated silica, copolymer of acrylic and itaconic acid, camphorquinone, dimethylaminobenzoate (-4), ethyl (dimethylamino)methacrylate, 2,6-di-tert-butyl-p-cresol*
Resin cement	Relyx Ultimate	3M Oral Care 4471448 /4788250	Base paste: Glass powder (65997-17-3), surface modified with 2- propenoic acid, 2 methyl-, 3-(trimethoxysilyl)propyl ester (2530-85-0) and phenyltrimethoxy silane (2996-92- ), bulk material; 2-propanoic acid, 2-methyl-, 1,1’ -[1- (hydroxymethyl)-1,2ethanediyl]ester, reaction products with 2-hydroxy-1,3-propanediyl dimethacrylate and phosphorus oxide; triethylene glycol dimethacrylate (tegdma), silane treated silica, oxide glass chemicals (non-fibrous) Catalyst paste: Glass powder (65997-17-3), surface modified with 2-propenoic acid, 2 methyl-3-(trimethoxysilyl)propyl ester (2530-85-0), bulk material; substituted dimethacrylate;2,4,6(1H,3H,5H)-pyrimidinetrione, 5-phenyl-1-(phenylmethyl)- calcium salt (2:1); 1,12-dodecane dimethycrylate; silane treated silica; sodium p-toluenesulfinate; 2-propenoic acid, 2-methyl-, [(3-methoxypropyl)imino]di-2,1-ethanediyl ester; calcium hydroxide; 2-propenoic acid, 2-methyl-, 2-[(2- hydroxyethyl)(3- methoxypropyl)amino]ethyl ester; titanium dioxide*

*The specific chemical identity and/or exact percentage (concentration) of this composition has been withheld as a trade secret.

### Ceramic Block Preparation

**Fig 1:**
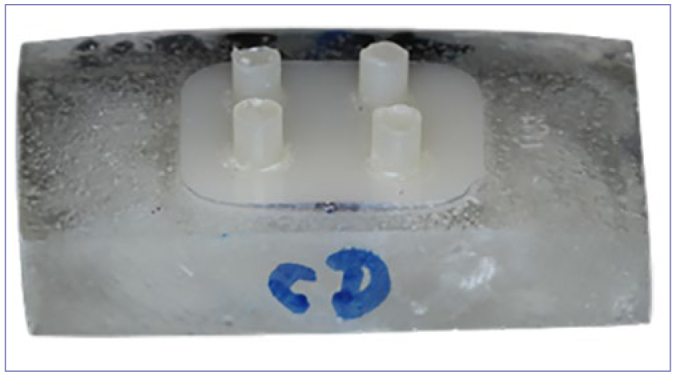
Ceramic block embedded in acrylic resin bearing 4 composite-cement cylinders.

Blocks for CAD/CAM made of lithium-silicate ceramics (LS, Celtra Duo, Dentsply Sirona; Konstanz, Germany), feldspathic ceramics (FD, Vita Mark II, VITA Zahnfabrik; Bad Säckingen, Germany) and polymer-infiltrated ceramics (PIC, Vita Enamic, VITA Zahnfabrik) were sectioned into 96 smaller blocks (10 x 12 x 2 mm) with double-sided diamond disks (22 mm x 0.15 mm, Dhpro; Paraná, Brazil) mounted on a micromotor straight handpiece. The block dimensions were measured with a digital caliper (150/6” MM Starrett 799A-6/150; mfc, location) and standardized with 200-, 400-, 600-, 800-, and 1200-grit SiC abrasive papers (3M Oral Care; St Paul, MN, USA) in a Politriz polishing machine (Labpol 8-12, Extec; Enfield, CT, USA) under water cooling. Then the ceramic blocks were placed in a silicone mold (Master-Talmax silicone; Curitiba, PR, Brazil) and embedded in acrylic resin (JET, Classic Dental Articles; Campo Limpo Paulista, SP, Brazil). The blocks of each ceramic were numbered, and simple randomization was performed to subdivide the blocks into 24 groups (4 ceramic blocks per group), according to the factors: “ceramic” (3 levels), “adhesive” (4 levels) and “aging” (2 levels). The experimental unit was the composite cement cylinder; n per group was 15 cylinders. On each ceramic block, 3 to 4 composite cement cylinders were made (Fig 1).

### Experimental Groups

All the ceramic blocks were cleaned in an ultrasonic bath with distilled water for 8 min (Cristófoli Equipamentos de Biossegurança; Campo Mourão, PR, Brazil). The adhesive area was delimited by a piece of adhesive tape bearing a perforation (Ø = 3 mm). Surface treatments were applied to the ceramics according to the experimental groups:

- HF + silane (control [Ctrl]): the ceramic surface was etched with 5% HF for the time appropriate to the ceramic type (LS: 30 s; FD and PIC: 60 s), followed by washing with a jet of water for 30 s and drying with a jet of air for 30 s. Then, silane (RelyX Ceramic Primer, 3M Oral Care) was applied for 1 min with a microbrush (Dentsply; Long Island City, NY, USA) and gentle air stream was applied to evaporate the solvent.
- HF + silane + 2-step conventional adhesive (2SC): the ceramic surface was etched and silanized as described above, followed by application of the conventional 2-step adhesive (Adper Single Bond 2, 3M Oral Care) with a microbrush for 10 s and dried with a gentle air stream for 5 s to evaporate the solvent.
- HF + silane + conventional 3-step adhesive (3SC): the ceramic surface was etched and silanized as described above, followed by application of the conventional 3-step adhesive adhesive only (3SC, Scotchbond Multi-Purpose Adhesive, 3M Oral Care) with a microbrush for 10 s and dried with a gentle air stream for 5 s to evaporate the solvent.
- HF + silane + 1-step universal adhesive (1SU): the ceramic surface was etched and silanized as described above, followed by application of the universal adhesive (1SU – Single Bond Universal, 3M Oral Care) with a microbrush for 20 s and dried with a gentle air stream for 5 s to evaporate the solvent.

### Preparation of Composite Cement Cylinders

A teflon matrix (Ultradent Jig, Ultradent; South Jordan, UT, USA) was adapted to the ceramic samples using a metal clamp. The composite cement base and catalyst pastes (Relyx Ultimate, 3M Oral Care) were mixed for 10 s and injected into the matrix perforation (Ø = 2 mm and height = 2 mm) using an injection syringe (Precision applicator, Maquira; Maringá, PR, Brazil) coupled to a needle-type tip no. 2 (Maquira), followed by light curing for 40 s (1200 mW/cm^2^, Radii Cal, SDI; Bayswater, Victoria, Australia). The irradiation of the polymerization lamp was checked with a radiometer before the preparation of the cylinders in each group. The device was then removed after the chemical polymerization time of the cement (10 min). Three to four cylinders of composite cement were made on each ceramic block (3-4 cylinders/ceramic block) (Fig 1), so that each group had four ceramic blocks and 15 cylinders of composite cement (n = 15; experimental unit: composite cement cylinder). The composite cement cylinders were inspected using magnification loupes to verify the presence of bubbles and defects that would indicate the exclusion of the cylinder from the sample.

### Long-term Aging

Half of the samples from each group were stored in distilled water at 37°C for 24 h (non-aged) and the other half underwent thermocycling (10,000 cycles of 5ºC and 55ºC, dwell time 30 s, transfer time 5 s; Nova ethics, São Paulo, SP, Brazil). They were then stored in distilled water at 37°C for 18 months (aged [A]).

### Shear Bond Strength Test

After each aging condition, the samples were subjected to shear bond strength testing in a universal testing machine (Microtensile OM150, Odeme Biotechnology; Luzerna, SC, Brazil). The samples were fixed in a metal clamp to position the composite cement/ceramic interface perpendicular to the horizontal plane (ISO 11405/2015). The load was applied to the base of the cylinder by a wire loop (Ø = 0.35 mm) at a crosshead speed of 1.0 mm/min until failure, with a load cell of 100 kgf. The formula R = F/A was used to calculate the shear bond strength, in which R is the bond strength (MPa), F is the force (N) recorded upon failure, and A is the cross-sectional interfacial area (3.14 mm^2^).

### Failure Analysis

**Fig 2:**
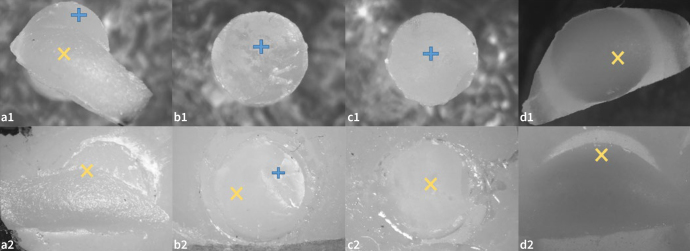
Optical stereomicroscope micrographs of the most frequent failure types between the ceramic and composite resin cement cylinder. a) Mixed 2: adhesive between the cement/ceramic interface + cohesive within the ceramic. b) Mixed 1: adhesive between the cement/ceramic interface + cohesive within the composite cement. c) Adhesive between the ceramic/cement interface. d) Cohesive within the ceramic. Ceramic (yellow X); composite cement (blue +).

The fractured surfaces (Fig 2) were examined under a stereomicroscope (Stereo Discovery V20, Zeiss; Göttingen, Germany) to classify failure type:^30^ a) adhesive at the ceramic/cement interface (A); b) cohesive within the ceramic (CE); c) mixed 1: adhesive at the cement/ceramic interface + cohesive within the composite cement (M1); and d) mixed 2: adhesive at the cement/ceramic interface + cohesive within the ceramic (M2).

Representative samples of the most frequent failure types were analyzed in a scanning electron microscope (SEM) at 50X, 70X, and 100X magnification (Hitachi TM 3000; Tokyo, Japan).

### Microscopic Analysis of the Adhesive Interface

Ceramic/cement/ceramic sandwich specimens were obtained for microscopic analysis of the adhesive interface. Two extra blocks of each ceramic considering the adhesive types were prepared and smoothed with sandpaper (200-, 400-, 600-, 800-, and 1200-grit). The surface treatments were performed and the adhesive applied according to the experimental group. Then, the treated ceramic blocks were bonded with a thin layer of composite cement. A load was applied (750 g) for 1 min and the excess composite cement was removed with a microbrush. Then, the cement was light cured (1200 mW/cm^2^, Radii Cal, SDI) for 40 s on each face of the block. The blocks were cut with a 2-sided diamond disk (Microdont, São Paulo, Brazil, no. 34.570) coupled to a straight piece and a micromotor (LB100 Beltec; São Paulo, Brazil) under air/water irrigation. The surface was smoothed with sandpaper (600-, 800-, 1200-, and 2000-grit), polished with <2-μm diamond polishing paste (Christensen Roder, Porto Alegre, RS, Brazil), and examined using SEM.

### Statistical Analysis

The experimental unit for statistical analysis was the composite cement cylinder. The normality and homoscedasticity of the shear bond strength data were analyzed using the Shapiro-Wilk and Levene tests, respectively. As the data were distributed normally and homogeneously, the results of shear bond strength testing were submitted to 3-factor ANOVA (ceramic, adhesive, and aging), followed by Tukey’s test (5%). Analyses were performed using the Jamovi Project software program (v.2.3, 2022, https://www.jamovi.org). Descriptive analysis of data from failure analysis and SEM of the adhesive interface was performed.

Weibull analysis was performed for each ceramic to investigate the bond strength reliability, using the Weibull Modulus (m), the characteristic strength (σ_0_), and a 95% confidence interval. Minitab software (v.17, 2013; State College, PA, USA) was used for this analysis.

## Results

### Shear Bond Strength

The bond strength data followed normal and homogeneous distribution (p<0.005). There were no pre-test failures. Three-way ANOVA revealed that all factors and the interaction between them significantly affected bond strength, with the exception of the interaction of the 3 factors “ceramics x adhesive x aging” (p = 0.2028).

LS_3SC (31.56 ± 6.27 MPa) and LS_1SU (31.18 ± 2.88 MPa) showed higher bond strength means for non-aged groups than LS_2SC (22.35 ± 5.99 MPa) and FD_2SC (24.10 ± 4.94 MPa), and were similar to the other groups. LS_Ctrl_A (30.30 ± 6.11 MPa), LS_3SC_A (27.74 ± 4.8 MPa), FD_Ctrl_A (26.86 ± 3.30 MPa), and FD_3SC_A (26.12 ± 4.55 MPa) showed higher bond strength means for aged groups than did LS_2SC_A (17.32 ± 5.86 MPa), and were statistically similar to most experimental groups (Table 2).

**Table 2:** Mean (MPa) and standard deviation of shear bond strength

Aging	Adhesive	Shear bond strength (MPa)		
		Lithium silicate	Feldspathic	Polymer-infiltrated ceramic
Non-aged	Ctrl	28.80 ± 4.29^ABa^	25.90 ± 5.13^Aa^	25.13 ± 4.15^Aa^
	2SC	22.35 ± 5.99^BCDa^	24.10 ± 4.94^Aa^	27.78 ± 5.73^Aa^
	3SC	31.56 ± 6.27^Aa^	26.19 ± 4.58^Aa^	26.47 ± 5.27^Aa^
	1SU	31.18 ± 2.88^Aa^	24.83 ± 4.27^Aa^	26.59 ± 6.35^Aa^
Aged (A)	Ctrl	30.30 ± 6.11^Aa^	26.86 ± 3.30^Aab^	21.98 ± 4.23^Ab^
	2SC	17.32 ± 5.86^Da^	21.99 ± 3.07^Aa^	21.44 ± 4.29^Aa^
	3SC	27.74 ± 4.89^ABCa^	26.12 ± 4.55^Aa^	22.51 ± 6.95^Aa^
	1SU	22.18 ± 7.74^CDa^	23.16 ± 4.89^Aa^	22.62 ± 5.48^Aa^

Different superscript capital letters indicate significant differences among groups within the columns (for each ceramic). Different superscript lowercase letters indicate significant differences among groups within the row (for each adhesive type). Ctrl: control; 2SC: 2-step conventional; 3SC: 3-step conventional; 1SU: 1-step-universal.

When comparing the control and the experimental groups for each ceramic, there was a significant difference only for the aged groups of LS. The LS_2SC_A and LS_1SU_A groups showed lower bond strength than did LS_Ctrl_A, which was similar to LS_3SC_A. However, there was no significant difference between the groups for FD and PIC. Moreover, when comparing the groups considering aging conditions, there was a significant decrease in the bond strength of the LS_1SU_A in comparison to the non-aged LS_1SU group. There was no significant difference between the aging conditions (non-aged and aged) of the other groups (Table 2).

### Weibull Analysis

The Weibull analysis for LS revealed that there were differences between the groups for both the Weibull modulus and characteristic strength. The LS_1SU (12.20) group showed the highest Weibull modulus, which was significantly higher than that of LS_2SC_A (2.82) and LS_1SU_A (3.15) groups. The LS_3SC (34.06) and LS_1SU (32.44) groups showed statistically higher characteristic strengths than did the LS_2SC (24.74) and LS_2SC_A (19.45) groups (Table 3).

**Table 3:** Weibull modulus (m), characteristic strength (σ_0_) and respective 95% confidence intervals (CI) for each ceramic

Aging	Adhesive	Weibull Modulus (m)	m 95% CI	Weibull characteristic strength (σ_0_) (MPa)	σ_0_ 95% CI (MPa)
**Lithium Silicate**					
Non-aged	Ctrl	7.88^ab^	5.59–11.12	30.49^αβ^	28.48–32.63
	2SC	3.74^ab^	1.98–7.07	24.74^βγ^	21.44–28.54
	3SC	5.56^ab^	3.34–9.24	34.06^α^	30.95–37.48
	1SU	12.20^a^	7.63–19.51	32.44^α^	31.05–33.89
Aged (A)	Ctrl	5.35^ab^	2.90–9.85	32.76^αβ^	29.65–36.18
	2SC	2.82^b^	1.46–5.45	19.45^γ^	16.11–23.49
	3SC	6.28^ab^	4.16–9.47	29.71^αβ^	27.29–32.34
	1SU	3.15^b^	2.02–4.88	24.70^αβ^	20.86–29.24
**Feldspathic**					
Non–aged	Ctrl	5.56^a^	3.63–8.46	27.97^αβ^	25.41–30.80
	2SC	5.71^a^	4.03–8.09	25.96^αβ^	23.63–28.52
	3SC	6.79^a^	4.74–9.72	27.94^α^	25.81–30.24
	1SU	6.52^a^	4.25–10.01	26.58^αβ^	24.49–28.84
Aged (A)	Ctrl	9.22^a^	5.75–14.77	28.26^α^	26.67–29.93
	2SC	8.41^a^	5.88–12.03	23.22^β^	21.78–24.75
	3SC	5.96^a^	3.21–11.06	28.09^α^	25.66–30.74
	1SU	5.93^a^	4.32–8.14	24.85^αβ^	22.67–27.24
**Polymer–infiltrated ceramic**					
Non–aged	Ctrl	6.75^a^	4.25–10.73	26.85^α^	24.81–29.05
	2SC	5.96^a^	4.36–8.15	29.82^α^	27.22–32.67
	3SC	5.75^a^	3.74–8.85	28.50^α^	25.98–31.26
	1SU	4.55^a^	2.97–6.97	29.06^α^	25.85–32.68
Aged (A)	Ctrl	5.40^a^	2.81–10.40	23.77^α^	21.53–26.27
	2SC	6.03^a^	4.07–8.93	24.11^α^	22.06–26.34
	3SC	2.55^a^	1.01–6.48	25.42^α^	20.38–31.71
	1SU	4.99^a^	3.54–7.05	24.51^α^	21.99–27.31

Different superscript lowercase letters indicate significant difference between groups for the Weibull modulus for each ceramic. Different superscript Greek letters indicate significant difference between groups for characteristic strength for each ceramic.

While no significant difference existed between the FD groups for Weibull modulus, there was a significant difference for characteristic strength. The FD_Ctrl_A (28.26), FD_3SC (27.94), and FD_3SC_A (28.09) groups showed significantly higher characteristic strength than did FD_2SC_A (23.22) (Table 3). There were no differences in PIC between groups for Weibull analysis and characteristic strength (Table 3).

### Failure Analysis

**Fig 3:**
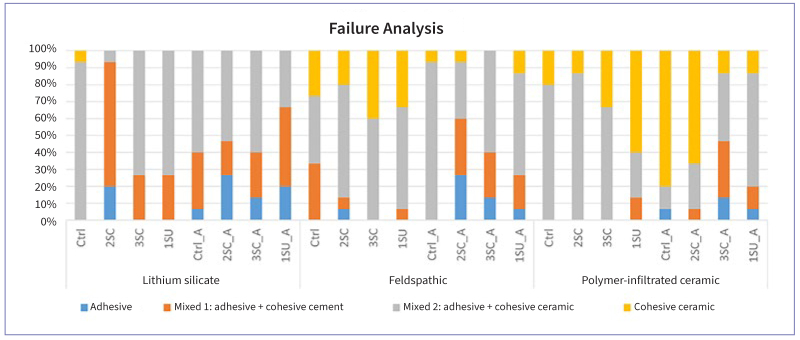
Distribution of failure types (%) after shear bond strength testing. Failure types: adhesive, mixed 1 (adhesive between the cement/ceramic interface + cohesive within the composite cement), mixed 2 (adhesive between the cement/ceramic interface + cohesive within the ceramic), cohesive in ceramic. Ctrl: control; 2SC: 2-step conventional adhesive; 3SC: 3-step conventional adhesive; 1SU: 1-step universal adhesive; NA: non-aged; A: aged.

**Fig 4:**
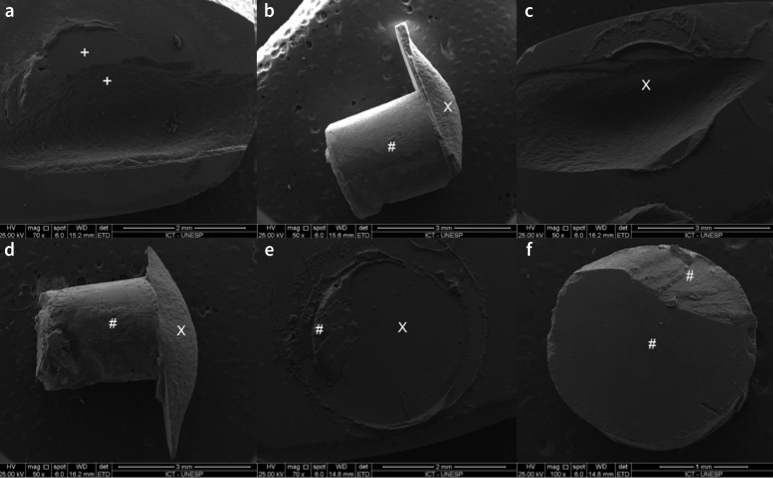
Representative SEM images of the most frequent failure types between ceramic and the composite-cement cylinder at 50X, 70X, and 100X magnification. Mixed 2: adhesive between the cement/ceramic interface + cohesive within the ceramic (a and b). Cohesive within the ceramic (c and d). Mixed 1: adhesive between the cement/ceramic interface + cohesive within the composite cement (e and f). Ceramic (X); composite cement (#).

The most prevalent failure type was mixed 2: adhesive + cohesive within ceramic (55.6%), followed by cohesive within ceramic (18.9%), mixed 1: adhesive + cohesive within composite cement (18.6%) and adhesive (6.9%). When analyzing ceramics, mixed 2 failure was the most frequent for all ceramics. Considering the type of adhesive, mixed 2 failure was also the most frequent in all groups (Figs 3 and 4).

### SEM Analysis of the Adhesive Interface

**Fig 5:**
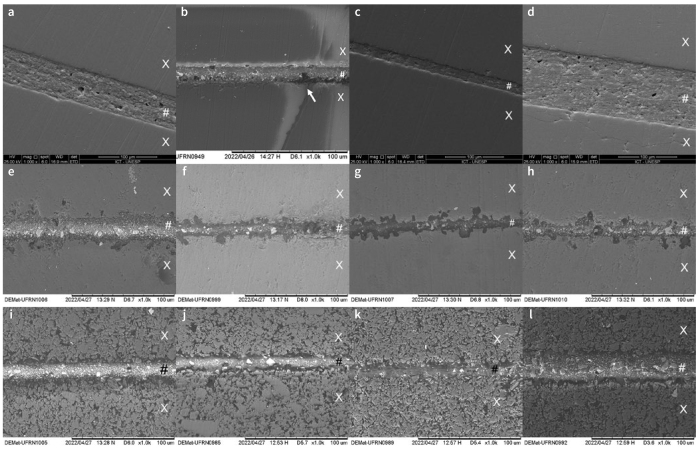
Representative SEM images of interfaces (all 1000X original magnification). a: adhesive interface of lithium silicate (LS) control ; b: LS with 1-step universal adhesive (1SU); c: LS with 2-step conventional adhesive (2SC); d: LS with 3-step conventional adhesive (3SC); e: feldspathic ceramic (FD) control; f: FD with 1SU; g: FD with 2SC; h: FD with 3SC; i: polymer-infiltrated ceramic (PIC) control; j: PIC with 1SU; k: PIC with 2SC; l: PIC with 3SC. Ceramic (X); composite cement (#).

In the adhesive interface between LS and the composite cement, it is possible to detect a homogeneous cementation line of ceramic irregularities by the composite cement in the Ctrl (Fig 5a), 1SU (Fig 5c) and 3SC (Fig 5d) groups. In contrast, defects (white arrow) were observed in the cementation line in group 2SC (Fig 5b). Homogeneous cementation was detected in all groups for FD (Fig 5e–h) and PIC (Fig 5i–l), which was even more evident for PIC.

## Discussion

The results of this study revealed that the application of an adhesive layer did not positively influence the bond strength between the ceramic and the composite cement, since no adhesive obtained bond strengths superior to that of the control group (without adhesive). The first hypothesis, which states that the adhesive type does not affect the bond strength between the tested ceramics and the composite cement, was partially rejected, considering that the adhesive type significantly influenced the adhesion for lithium-silicate, but did not affect polymer-infiltrated and feldspathic ceramics.

These results corroborate the findings of the systematic review and meta-analysis by Nogueira et al,^19^ who concluded that the an adhesive layer should not be applied on etched and silanized ceramics, since the meta-analysis comparing the bond strength of groups with and without adhesive did not favor application of the adhesive. This systematic review and meta-analysis included studies with and without aging; however, subgroup analyses were not performed to elucidate whether the type of adhesive affects the results. Other laboratory studies that evaluated the effect of applying an adhesive and artificial aging also indicated similar results between the groups with and without application of the adhesive,^5,17,23,31^ or that adhesive application negatively influenced ceramic/composite cement adhesion.^20^

Another important finding of the present study was the lower bond strength of the aged LS groups with 2SC and 1SU adhesive application compared to the control. It was also observed that 2SC and 1SU yielded less reliable adhesion and exhibited more heterogeneous behavior, since these adhesives had reduced Weibull modulus and characteristic strengths. These findings may be related to the physicochemical characteristics of these adhesives. They may have a higher degree of hydrophilicity because they contain hydrophilic monomers and solvents, which may contribute to greater water sorption, hydrolytic degradation, and hygroscopic expansion of the adhesive interface after artificial aging.^6,13^ These characteristics may also have promoted flaw formation in the cementation line in lithium-silicate ceramic samples to which 2SC adhesive was applied, as reflected in the SEM analysis of the adhesive interface. This may have contributed to lower bond strength in this group compared to the control. Brito et al^4^ observed that applying an additional layer of hydrophobic adhesive in combination with a universal adhesive improved adhesion to dentin after aging. This finding reinforces the crucial role of the physical and chemical characteristics of the adhesive in maintaining adhesion stability over time.

Previous studies have reported different findings from this study. Murillo-Gómez et al^17^ and Romanini-Junior et al^23^ reported that a 2SC adhesive showed bond strength comparable to that of the control group (without adhesive) after aging on lithium-disilicate samples. Passos et al^18^ detected a considerable decrease in the bond strength of the feldspathic ceramic samples treated with a 3SC adhesive after aging. This divergence in results may be related to methodological differences between studies, which include variations in the ceramic type, silane composition (presence of 10-MDP), adhesive brand, composite cement type, bond strength test, and artificial aging method, among others.^5,19^

The second hypothesis, that the ceramic type does not influence the bond strength to composite cement, was rejected. The ceramics investigated in this study (LS, FD, and PIC) have different microstructural characteristics, endowing them with different surface characteristics after acid etching and different interaction with the adhesives and composite cement.^31^ Thus, although FD and PIC presented lower bond strengths than LS, they showed less variation in bond strengths, considering the different types of adhesives tested and aging conditions. The longer etching time indicated for FD and PIC (60 s) may promote better micromechanical interlocking between these ceramics and the composite cement, making them less influenced by the effect of adhesive type and aging.

Furthermore, the polymeric network in the PIC’s microstructure may have promoted strong adhesion to the composite cement,^4^ thus contributing to the higher frequency of cohesive failures in this ceramic. This indicates that the adhesion between this ceramic and composite cement was higher than the mechanical strength of the ceramic, causing failure of the ceramic substrate.^28^ This is corroborated by the microscopic analysis of the adhesive interface, which revealed a homogeneous imbrication between the composite cement and the PIC. Similar results were reported by Hu et al,^11^ who detected a higher percentage of cohesive ceramic failures for polymer-infiltrated ceramics and feldspathic ceramics than for lithium-silicate, which showed higher bond strengths. The higher mechanical strength of the lithium-silicate surpassed the bond strength at the adhesive interface, resulting in a lower frequency of cohesive ceramic failure for this ceramic.

Another factor investigated in this study was the effect of long-term aging on the bond strength between ceramics and composite cement. The samples were submitted to two artificial aging methods to expose them to longer and more severe aging, thus better imitating the challenges inherent in the oral environment. Most studies in the literature performed thermocycling for up to 10,000 cycles or storage in water for up to 12 months.^16,19^ Studies that subjected cemented ceramic samples (focussing on the bonded area) to higher numbers of thermocycles or longer storage periods in water are scarce in the literature.^2^

Thus, the third hypothesis, that aging does not decrease the bond strength between the tested ceramics and composite cement, was partially accepted. Despite the fact that the aging factor was significant, aging only negatively influenced adhesion in LS samples to which 1SU adhesive was applied. There was also a considerable decrease in the characteristic strength in the LS_1SU aged group. This decrease may be related to higher hydrophilicity at the adhesive interface in this group, which can contribute to greater water sorption and hydrolytic degradation at the adhesive interface.^6,13^

Although this study’s samples were submitted to two types of artificial aging, in addition to a longer storage period in water than reported by most studies,^5,19^ stable adhesion was observed in most experimental groups. Adhesion to silica-based ceramics is considered one of the great advantages of this material due to its sensitivity to acid etching. Furthermore, no adhesive, regardless of physicochemical characteristics, showed more stable adhesion than the control, indicating that adhesive application is not necessary for the long-term success of ceramic restorations. Moreover, clinical studies^14,15^ have shown that surface treatment using acid and silane etching, with or without adhesive application, has produced satisfactory and long-lasting adhesion.

Thus, considering that the tested adhesives have different components, it can be assumed that they can influence the physicochemical characteristics of a ceramic surface, as well as the adhesive interface that will be exposed to the oral environment. UNV adhesive contains different components, which can make it more hydrophilic than 3SC adhesive, which is composed of pure adhesive (comprising the bonding agent with no additional components).^6,20,29^

Therefore, the results of this study demonstrate that the application of an adhesive after etching and silanization of LS, FD, and PIC ceramics is not mandatory for the success and longevity of adhesion. Regardless of adhesive type, no group in which adhesive was applied showed improved ceramic adhesion to composite cement compared to the groups without application of the adhesive. Furthermore, the findings suggest that the use of universal adhesive and conventional two-step adhesive in the treatment of LS ceramics may even be disadvantageous, as bond strength in these groups was lower than in the group without adhesive. Additional laboratory studies are important to investigate the long-term interaction of adhesives with different composite cement types. Moreover, clinical studies evaluating the effect of adhesive application on the longevity and success of glass-ceramic restorations are important to understand and improve the adhesive performance of indirect ceramic restorations and minimize adhesion-related failures. A limitation of this study was the use of products by only one manufacturer, so the extrapolation of the results to other products must be done cautiously.

## Conclusion

The application of an adhesive layer after silanization of silica-based ceramics does not improve the bond strength to composite cement for all tested ceramics, regardless of the adhesive type. In addition, the application of 1-step universal and 2-step conventional adhesive to lithium-silicate ceramics should be avoided, as they reduce the bond strength in relation to the control.

## Clinical relevance:

The application of an adhesive layer after etching (HF) and silanization of the silica-based ceramics tested does not improve the bond strength durability of composite cement; thus, it is not recommended.
